# Risk Factors Associated With Inferior Alveolar and Lingual Nerve Injury Following Surgical Extraction of Impacted Mandibular Third Molars: A Retrospective Cohort Study

**DOI:** 10.7759/cureus.113638

**Published:** 2026-07-30

**Authors:** Deepak Yadav, Ravi Ranjan, Mohammad Muneeb Mubashir, Monika Koul, Sanpreet S Sachdev, Poorwa Sharma

**Affiliations:** 1 Department of Oral and Maxillofacial Surgery, Faculty of Dental Sciences, Shree Guru Gobind Singh Tricentenary University, Gurugram, IND; 2 Department of Orthodontics, Geeta Devi's Multi-Speciality Dental Clinics, Patna, IND; 3 Department of Dentistry, Government Medical College, Handwara, IND; 4 Department of Orthodontics, K. M. Shah Dental College and Hospital, Sumandeep Vidhyapeeth (Deemed to be University), Vadodara, IND; 5 Department of Oral Pathology and Microbiology, Bharati Vidyapeeth (Deemed to be University) Dental College and Hospital, Navi Mumbai, IND; 6 Department of Oral and Maxillofacial Surgery, Geetanjali Dental and Research Institute, Udaipur, IND

**Keywords:** impacted mandibular third molar, inferior alveolar nerve injury, lingual nerve injury, neurosensory deficit, risk factors

## Abstract

Introduction: Injury to the inferior alveolar and lingual nerves is a recognized complication of surgical extraction of impacted mandibular third molars and may result in temporary or permanent neurosensory disturbances. The identification of patient-, radiographic-, and procedure-related risk factors is essential for optimizing surgical planning and minimizing postoperative complications. This study aimed to evaluate the incidence of postoperative inferior alveolar and lingual nerve injuries following impacted mandibular third molar surgery and to identify the demographic, radiographic, and intraoperative factors associated with their occurrence.

Methodology: This retrospective cohort study included 135 patients (165 impacted mandibular third molars) who underwent surgical extraction. Demographic characteristics, radiographic findings, and intraoperative variables were retrieved from the clinical records. Generalized estimating equations were used to account for the clustering arising from bilateral impactions. Variables that were significant on univariate analysis were entered into a multivariable model to identify independent predictors of postoperative nerve injury.

Results: Postoperative neurosensory deficits occurred in 12 (7.3%) molars, including 9 (5.5%) transient and 3 (1.8%) permanent nerve injuries. Pell and Gregory Class C impactions, horizontal angulation, and radiographic overlap with the inferior alveolar canal were identified as independent predictors of nerve injury in patients aged ≥ 35 years. Prolonged surgical duration and extensive bone removal were also significantly associated with a higher incidence of postoperative neurosensory deficits, whereas root morphology and coronectomy were not significantly associated.

Conclusions: Postoperative nerve injury following mandibular third molar surgery is uncommon and transient. Advanced age, deep impactions, horizontal angulation, and close radiographic proximity to the inferior alveolar canal significantly increase the risk of neurosensory complications. Careful preoperative assessment and meticulous surgical planning may facilitate risk stratification and contribute to improved surgical outcomes.

## Introduction

Surgical removal of impacted mandibular third molars is among the most frequently performed procedures in oral and maxillofacial surgery [1]. Although the procedure is generally safe and predictable, it is associated with several postoperative complications including pain, swelling, trismus, infection, alveolar osteitis, hemorrhage, and neurosensory disturbances [2,3]. Injury to the inferior alveolar nerve (IAN) and lingual nerve (LN) remains one of the most clinically significant complications because of its potential to produce altered sensation, numbness, paresthesia, dysesthesia, or loss of function involving the lower lip, chin, mandibular teeth, and tongue [4]. While most nerve injuries resolve spontaneously within weeks or months, a small proportion may persist permanently, adversely affecting mastication, speech, oral function, and the overall quality of life.

The occurrence of nerve injury following third molar surgery is multifactorial and influenced by patient-related, radiographic, and surgical variables. Increasing patient age, depth of impaction, tooth angulation, root morphology, and the anatomical relationship between the impacted tooth and inferior alveolar canal have all been implicated as potential risk factors [5,6]. Radiographic assessment using panoramic radiography and cone-beam computed tomography plays an essential role in identifying high-risk cases and guiding surgical planning [7,8]. Intraoperative factors, including surgical duration, extent of bone removal, tooth sectioning, and complexity of extraction, may further influence the likelihood of postoperative neurosensory complications [5,6]. Nevertheless, the relative contributions of these factors remain inconsistent across published studies, owing to differences in study design, patient populations, and analytical approaches.

Accurate identification of the independent predictors of nerve injury is essential for improving preoperative risk stratification, patient counselling, informed consent, and surgical decision-making. Furthermore, understanding these predictors may facilitate the adoption of alternative treatment strategies, such as coronectomy or modified surgical techniques, in patients at increased risk of nerve damage [9].

The present study aimed to determine the incidence of postoperative neurosensory injury, defined as the occurrence of either IAN or LN injury following surgical extraction of impacted mandibular third molars, and to identify the demographic, radiographic, and intraoperative factors associated with this composite outcome. Because of the relatively low frequency of postoperative nerve injuries, IAN and LN injuries were analyzed as a combined postoperative neurosensory outcome. The primary objective of this study was to determine the incidence of postoperative IAN and LN injury following surgical extraction of impacted mandibular third molars. The secondary objective was to identify the demographic, radiographic, and intraoperative factors associated with postoperative nerve injury. Specifically, the study evaluated the associations of patient age, sex, impaction characteristics, radiographic proximity to the inferior alveolar canal, surgical duration, bone removal, root morphology, and coronectomy with postoperative neurosensory deficits, and identified independent predictors using multivariable statistical analysis.

## Materials and methods

This retrospective cohort study was conducted in the Department of Oral and Maxillofacial Surgery at Geetanjali Dental and Research Institute, Udaipur, India, in June 2023 using clinical and radiographic records of patients who underwent surgical extraction of impacted mandibular third molars at the department between January 2021 and April 2023. Institutional Ethics Committee approval was obtained before commencement of the study (IEC no: GU/IREC/EC/2023/1876, May 24, 2023). Owing to the retrospective nature of the study, the requirement for informed consent was waived by the Institutional Ethics Committee.

Sample size estimation

Sample size estimation was performed a priori using G*Power software version 3.1.9.7 (Heinrich Heine University Düsseldorf, Düsseldorf, Germany). Based on a previously reported postoperative nerve injury prevalence of 7.2%, a two-tailed z-test for independent proportions was applied with 80% statistical power, a two-sided alpha level of 0.05, and 5% margin of error [10]. The initial calculation yielded a minimum of 114 impacted mandibular third molars. To account for the inclusion of patients with bilateral impactions and the resulting intra-patient correlation, a conservative design effect was incorporated, which increased the sample size to 130 molars. An additional 25% inflation was applied to ensure adequate power for multivariable regression analyses and to compensate for incomplete records, resulting in a final target sample of 165 impacted mandibular third molars.

Inclusion and exclusion criteria

Patient records were screened consecutively according to predefined eligibility criteria. Patients aged 18 years or older who underwent surgical extraction of at least one impacted mandibular third molar between January 2020 and December 2024 were eligible for inclusion. Only patients with complete preoperative clinical records, diagnostic panoramic radiographs, operative notes, and postoperative neurosensory follow-up documentation were included. Both unilateral and bilateral mandibular third molar impactions were eligible. Patients were excluded if they had pre-existing IAN or LN dysfunction, previous mandibular surgery, mandibular fractures, craniofacial anomalies, pathological lesions associated with impacted third molars, incomplete clinical or radiographic records, or inadequate postoperative follow-up for assessment of neurosensory recovery.

Assessment and diagnosis of nerve injury

The primary outcome was the occurrence of postoperative IAN or LN injury. Postoperative neurosensory evaluation was performed by a blinded examiner who was not involved in the radiographic assessment. Nerve injury was diagnosed based on standardized postoperative clinical examination and documentation of subjective and objective neurosensory disturbances involving the distribution of the IAN or LN, including altered sensation, paresthesia, hypoesthesia, dysesthesia, or anesthesia affecting the lower lip, chin, mandibular dentition, or tongue. Clinical findings were recorded during routine postoperative follow-up visits. Neurosensory deficits that resolved completely during follow-up were classified as transient, whereas symptoms persisting for more than six months were classified as permanent nerve injuries.

Data evaluation

Demographic variables, including age and sex, were extracted from patient records. Radiographic evaluation of all preoperative panoramic radiographs was performed independently by two specialists (DY and MMM), who were affiliated with two different institutions and blinded to the postoperative neurosensory outcomes. Before commencement of the study, both examiners underwent calibration using a representative set of panoramic radiographs. To assess the reproducibility of the radiographic interpretation, 30 randomly selected radiographs were re-evaluated independently after a two-week interval. Inter-examiner and intra-examiner reliabilities were assessed using Cohen's kappa coefficient (κ), which demonstrated excellent agreement (inter-examiner κ = 0.89; intra-examiner κ = 0.92). Any discrepancies in radiographic interpretation were resolved by consensus.

Postoperative clinical examination and assessment of inferior alveolar and lingual nerve function were performed by another specialist (RR) who was blinded to the radiographic assessments. Preoperative panoramic radiographs were used for radiographic assessment. The depth of impaction and ramus relationship were classified according to the Pell and Gregory classification [11], whereas tooth angulation was categorized using Winter's classification [12]. The radiographic relationship between the roots of the impacted mandibular third molar and the inferior alveolar canal was evaluated and categorized as either overlapping or non-overlapping. Additional intraoperative variables retrieved from the operative records included surgical duration (<30 minutes or ≥30 minutes), extent of bone removal (minimal or extensive buccal guttering), root morphology (single/fused or divergent/curved roots), and whether coronectomy was performed.

Follow-up

Patients underwent routine postoperative follow-up according to departmental clinical protocols. Neurosensory status was assessed during postoperative visits until complete recovery or for a minimum of six months after surgery. Patients demonstrating persistent neurosensory deficits beyond the six-month follow-up period were classified as having permanent nerve injury.

Data analysis

Data were then statistically analyzed by a statistician who was provided with coded data (MK) (IBM SPSS Statistics for Windows, version 26.0, IBM Corp., Armonk, NY) and STATA version 17.0 (StataCorp LLC, College Station, TX). Continuous variables were summarized as mean ± standard deviation, whereas categorical variables were expressed as frequencies and percentages. As bilateral impacted third molars from the same patient were included, the observations were not statistically independent. Therefore, all univariate and multivariable analyses were performed using generalized estimating equations (GEEs) with a binomial distribution, logit link function, and exchangeable working correlation structure to account for within-patient clustering. Variables demonstrating a univariate association with a *P* ≤ 0.20 were entered into the multivariable GEE model to identify independent predictors of postoperative nerve injury. Model adequacy was assessed using quasi-likelihood under the independence criterion (QIC). Adjusted odds ratios (aORs) with corresponding 95% confidence intervals (CIs) were calculated for all independent predictors, and a two-tailed *P* < 0.05 was considered statistically significant for all analyses.

## Results

A total of 135 patients with 165 impacted mandibular third molars were included in the study, of which 30 (22.2%) presented with bilateral impactions. The mean patient age was 28.4 ± 8.2 years, and females accounted for 85 (63.0%) of the study population. According to the Pell and Gregory classification, 70 (42.5%) molars were classified as Class B, 55 (33.3%) as Class C, and 40 (24.2%) as Class A. Class I ramus relationships were observed in 85 (51.5%) of the molars. Based on Winter's classification, mesioangular impactions were the most common, followed by distoangular and horizontal. Radiographic overlap between the impacted roots and inferior alveolar canal was observed in 60 molars (36.4%). Overall, postoperative neurosensory deficits occurred in 12 (7.3%) molars, including 9 (5.5%) transient injuries and 3 (1.8%) permanent nerve injuries (Table 1).

**Table 1:** Baseline demographic and radiographic characteristics of the study population (n = 165 impacted mandibular third molars). Data are presented as mean ± standard deviation or *n* (%). Pell and Gregory, Pell and Gregory classification; IAN, inferior alveolar nerve; LN, lingual nerve; SD, standard deviation

Variable	Category	Value
Patient-level demographics	Total patients/impacted tooth	135/165
	Age (years), mean ± SD	28.4 ± 8.2
	Sex, female, *n* (%)	85 (63.0)
	Bilateral impactions, *n* (%)	30 (22.2)
Pell-Gregory depth, *n* (%)	Class A (High)	40 (24.2)
	Class B (Moderate)	70 (42.5)
	Class C (Deep)	55 (33.3)
Pell-Gregory ramus, *n* (%)	Class I (Adequate space)	85 (51.5)
	Class II (Reduced space)	50 (30.3)
	Class III (No space)	30 (18.2)
Winter’s angulation, *n* (%)	Mesioangular	50 (30.3)
	Vertical	40 (24.2)
	Distoangular	45 (27.3)
	Horizontal	30 (18.2)
Radiographic root proximity, *n* (%)	Overlap with IAN canal (Yes)	60 (36.4)
	Overlap with IAN canal (No)	105 (63.6)
Nerve injury outcome, *n* (%)	Total Nerve Injuries (*n*, %)	12 (7.3)
	Transient IAN/LN injury	9 (5.5)
	Permanent IAN/LN injury	3 (1.8)
Surgical duration, *n* (%)	< 30 minutes	105 (63.6)
	≥ 30 minutes	60 (36.4)
Bone removal, *n* (%)	Minimal (Envelope flap)	90 (54.5)
	Extensive (Buccal guttering)	75 (45.5)
Root configuration, *n* (%)	Single/fused roots	110 (66.7)
	Divergent/curved roots	55 (33.3)
Coronectomy performed, *n* (%)	No	150 (90.9)
	Yes (due to high risk)	15 (9.1)

Univariate GEE analysis demonstrated that patients aged ≥35 years had the highest incidence of postoperative nerve injury, with 5 (16.7%) injuries among 30 molars, compared with 3 (3.8%) injuries among 80 molars in patients younger than 25 years (*P* = 0.039). Pell and Gregory Class C impactions showed the greatest frequency of nerve injury, with 7 (12.7%) injuries among 55 molars, whereas Class A impactions demonstrated only 1 (2.5%) injury among 40 molars (*P* = 0.025). Horizontal impactions exhibited the highest injury rate, followed by distoangular, mesioangular, and vertical impactions (*P* = 0.011). Radiographic overlap with the inferior alveolar canal was associated with a significantly greater incidence of postoperative nerve injury, occurring in 8 (13.3%) of 60 overlapped molars than in 4 (3.8%) of 105 molars without overlap (*P* = 0.009). Neither sex (*P* = 0.622) nor the Pell-Gregory ramus relationship (*P* = 0.163) demonstrated a statistically significant association with nerve injury (Table 2).

**Table 2:** Univariate analysis of factors associated with postoperative nerve injury using generalized estimating equations (GEEs). Data are presented as *n* (%) or odds ratio (OR) with 95% confidence interval (CI). Wald χ² statistics were obtained from the GEE model with a binomial distribution, logit link function, and exchangeable correlation structure using robust standard errors. *P < 0.05 was considered statistically significant. IAN, inferior alveolar nerve

Variable	Category	Total molars (*N* = 165), *n* (%)	Injury, *n* (%)	Unadjusted OR (95% CI)	Wald χ²	*P*-value
Age group, years	<25	80 (48.5)	3 (3.8)	Reference	4.27	0.039*
	25-34 years	55 (33.3)	4 (7.3)	1.99 (0.92-4.30)		
	≥35 years	30 (18.2)	5 (16.7)	4.82 (1.21-19.20)		
Sex	Male	80 (48.5)	5 (6.3)	Reference	0.24	0.622
	Female	85 (51.5)	7 (8.2)	1.31 (0.46-3.78)		
Pell-Gregory depth	Class A	40 (24.2)	1 (2.5)	Reference	5.01	0.025*
	Class B	70 (42.4)	4 (5.7)	2.35 (0.78-7.10)		
	Class C	55 (33.3)	7 (12.7)	5.87 (1.42-24.20)		
Winter’s angulation	Mesioangular	50 (30.3)	2 (4.0)	Reference	6.52	0.011*
	Vertical	40 (24.2)	1 (2.5)	0.62 (0.10-3.90)		
	Distoangular	45 (27.3)	5 (11.1)	3.00 (1.10-8.20)		
	Horizontal	30 (18.2)	4 (13.3)	3.82 (1.09-13.40)		
IAN canal overlap	No	105 (63.6)	4 (3.8)	Reference	6.82	0.009*
	Yes	60 (36.4)	8 (13.3)	3.92 (1.47-10.40)		
Pell-Gregory ramus	Class I	85 (51.5)	5 (5.9)	Reference	1.95	0.163
	Class II	50 (30.3)	4 (8.0)	1.38 (0.68-2.80)		
	Class III	30 (18.2)	3 (10.0)	1.77 (0.72-4.35)		

Despite identical marginal distributions for IAN canal overlap and surgical duration, cross-tabulation confirmed that these variables were not completely concordant at the individual molar level (Table 3).

**Table 3:** Cross-tabulation for IAN overlap and time required for surgery. Data presented as frequency and percentage. IAN, inferior alveolar nerve

IAN canal overlap	Surgery <30 minutes, *n* (%)	Surgery ≥30 minutes, *n* (%)	Total
No (*n* = 105)	85 (81.0%)	20 (19.0%)	105 (100.0%)
Yes (*n* = 60)	20 (33.3%)	40 (66.7%)	60 (100.0%)

Multivariate GEE analysis identified several independent predictors of postoperative nerve injury. Patients aged ≥35 years had significantly higher odds of developing nerve injury than those aged <25 years (aOR = 4.50, 95% CI: 1.20-16.80, *P* = 0.025). Pell and Gregory Class C impactions independently increased the risk of postoperative nerve injury (aOR = 5.10, 95% CI: 1.13-23.00, *P* = 0.030). Horizontal impactions were also independently associated with a significantly greater risk than mesioangular impactions (aOR = 3.80, 95% CI: 1.10-13.20, *P* = 0.036). Radiographic overlap with the inferior alveolar canal remained a strong independent predictor (aOR = 4.20, 95% CI: 1.30-13.50, *P* = 0.013), whereas sex was not significantly associated with postoperative nerve injury (*P* = 0.725). The final multivariable model demonstrated a satisfactory fit, with a QIC value of 85.4 (Table 4).

**Table 4:** Multivariable generalized estimating equations (GEEs) analysis identifying independent predictors of postoperative nerve injury. Data are presented as adjusted odds ratios (OR) with 95% confidence intervals (CI); quasi-likelihood under the independence model criterion (QIC) was used to assess model fit (QIC = 85.4); Wald χ² statistics were obtained from the multivariable GEE model with a binomial distribution, logit link function, and exchangeable correlation structure using robust standard errors. *P < 0.05 was considered statistically significant.

Independent variable	Reference/Comparison	Adjusted odds ratio (aOR)	95% CI	Wald χ²	*P*-value
Age ≥ 35 years	Age < 25 years	4.50	1.20-16.80	5.01	0.025*
Age 25-34 years	Age < 25 years	1.85	0.80-4.25	2.10	0.147
Pell-Gregory class C	Class A	5.10	1.13-23.00	4.70	0.030*
Pell-Gregory class B	Class A	2.10	0.65-6.80	1.52	0.218
Horizontal angulation	Mesioangular	3.80	1.10-13.20	4.40	0.036*
Distoangular angulation	Mesioangular	2.90	0.98-8.60	3.70	0.054
IAN canal overlap (Yes)	No overlap	4.20	1.30-13.50	6.20	0.013*
Sex (Female)	Male	1.20	0.40-3.50	0.12	0.725

Subgroup analysis showed in Table 5 that procedures lasting ≥30 min resulted in postoperative nerve injury in 8 (13.3%) of 60 molars compared with 4 (3.8%) of 105 molars undergoing shorter procedures (aOR = 3.85, 95% CI: 1.45-10.20, *P* = 0.007). Extensive bone removal through buccal guttering was associated with nerve injury in 9 (12.0%) of 75 molars compared to 3 (3.3%) of 90 molars treated with minimal bone removal (aOR = 4.00, 95% CI: 1.10-14.50, *P* = 0.032). Divergent or curved roots demonstrated nerve injury in 7 (12.7%) of 55 molars, whereas 5 (4.5%) of 110 molars with single or fused roots developed injury; however, this difference was not statistically significant (*P* = 0.059). Coronectomy was performed in 15 high-risk molars and was associated with nerve injury in 1 (6.7%) case compared with 11 (7.3%) injuries among 150 conventionally extracted molars, with no statistically significant difference (*P* = 0.884).

**Table 5:** Association between intraoperative factors and postoperative nerve injury. Data are presented as *n* (%) or adjusted odds ratio (aOR) with 95% confidence interval (CI). Wald χ² statistics were obtained from the GEE model with a binomial distribution, logit link function, and exchangeable correlation structure using robust standard errors. Coronectomy was selectively performed in high-risk cases; therefore, the absence of a statistically significant association is likely attributable to selection bias resulting from its use as a protective surgical strategy in anatomically challenging cases. *P < 0.05 was considered statistically significant. GEE, generalized estimating equation

Intraoperative variable	Category	Total molars (*N*)	Nerve injury, *n *(%)	Adjusted OR (95% CI)	*P*-value
Surgical duration	<30 minutes	105	4 (3.8%)	Reference	0.007*
	≥30 minutes	60	8 (13.3%)	3.85 (1.45-10.20)	
Bone removal	Minimal (Envelope flap)	90	3 (3.3%)	Reference	0.032*
	Extensive (Buccal guttering)	75	9 (12.0%)	4.00 (1.10-14.50)	
Root configuration	Single/fused roots	110	5 (4.5%)	Reference	0.059
	Divergent/curved roots	55	7 (12.7%)	3.10 (0.95-10.10)	
Coronectomy performed	No	150	11 (7.3%)	Reference	0.884
	Yes (due to high risk)	15	1 (6.7%)	0.91 (0.25-3.30)	

## Discussion

The present retrospective cohort study evaluated the incidence and determinants of IAN and LN injuries following surgical extraction of impacted mandibular third molars. The overall incidence of postoperative neurosensory deficits was 7.3%, with 5.5% of cases being transient and 1.8% resulting in permanent nerve injury. These findings are consistent with the literature reporting nerve injury rates ranging from approximately 0.5% to 8% for the IAN and 0.1% to 2% for LN injuries, depending on patient characteristics, surgical complexity, and follow-up duration [4,10]. The predominance of transient over permanent injuries observed in the present study supports the concept that most postoperative neurosensory disturbances represent temporary neuropraxia caused by surgical manipulation, compression, or traction of the nerve, with gradual recovery occurring through nerve regeneration [13,14].

Age emerged as an independent predictor of postoperative nerve injury, with patients aged ≥ 35 years demonstrating significantly greater odds of neurosensory complications than younger individuals. Similar observations have been reported in previous studies, which attributed the increased risk in older patients to greater bone density, reduced periodontal ligament space, complete root maturation, and stronger ankylosis between the tooth and the surrounding bone [6,15]. These anatomical changes increase surgical difficulty, often necessitating more extensive bone removal and prolonged instrumentation, thereby increasing the likelihood of direct or indirect nerve trauma [16]. The present multivariate analysis indicates that age remains an independent contributor, even after accounting for other important variables.

Depth of impaction is another important determinant of nerve injury. Pell and Gregory Class C impactions exhibited a nearly five-fold increase in the odds of postoperative neurosensory deficits compared with Class A impactions. Similar findings have been consistently documented in previous investigations, in which deeply impacted third molars were associated with greater surgical difficulty and a closer anatomical relationship with the mandibular canal [1,6,17]. Deep impactions require a wider flap reflection, greater bone removal, and more extensive tooth sectioning, thereby increasing the probability of nerve compression or mechanical injury. Although some studies have suggested that limited retromolar space, represented by advanced Pell and Gregory ramus classes, may increase surgical difficulty and the risk of postoperative nerve injury, no significant association with ramus classification was observed in the present study [6,16]. This finding suggests that the ramus relationship alone may be insufficient for predicting neurosensory complications when other anatomical and surgical factors are considered.

Tooth angulation also significantly influenced the postoperative outcomes. Horizontal impactions demonstrated the highest independent risk of nerve injury, whereas distoangular impactions showed a borderline association. Comparable findings have been reported previously, where horizontally impacted molars frequently require extensive bone guttering and complex tooth sectioning because of their unfavorable orientation. Increased manipulation during luxation may transmit greater pressure to the inferior alveolar canal, particularly when the root apices lie in close proximity to the nerve [18,19].

Radiographic overlap between the impacted roots and inferior alveolar canal was one of the strongest independent predictors of nerve injury in the present study. Patients with radiographic overlap demonstrated more than fourfold higher odds of postoperative neurosensory deficits than those without overlap. This observation agrees with numerous radiographic studies demonstrating that close approximation between the roots and mandibular canal substantially increases the possibility of direct nerve trauma during extraction [7,8,20]. Panoramic radiographic signs, such as interruption of the canal cortex, diversion of the canal, or darkening of the roots, have been shown to correlate with intimate nerve contact, while cone-beam computed tomography provides superior three-dimensional assessment for surgical planning [7,21]. These findings reinforce the importance of comprehensive preoperative imaging when conventional radiographs indicate a high-risk relationship.

Among intraoperative variables, prolonged surgical duration and extensive bone removal were significantly associated with postoperative nerve injury. Longer procedures generally reflect greater surgical difficulty and prolonged tissue manipulation, increasing the likelihood of mechanical compression, thermal injury, and postoperative inflammation around the nerve [1,22]. Similarly, extensive buccal guttering may expose the mandibular canal more closely and increase the risk of inadvertent trauma. Although divergent or curved root morphology demonstrated a higher incidence of nerve injury [23], the association did not reach statistical significance, possibly because of the relatively limited number of affected cases. Similarly, coronectomy did not significantly reduce nerve injury in this cohort. This finding should be interpreted cautiously because coronectomy was selectively performed in patients considered to be at the highest risk of nerve injury, introducing selection bias and limiting direct comparisons with conventional extraction.

Clinical implications

Our findings have several important clinical implications. Preoperative identification of high-risk patients based on age, impaction depth, tooth angulation, and radiographic proximity to the inferior alveolar canal enables clinicians to provide more accurate risk assessments and informed consent. Patients presenting with multiple high-risk features may benefit from advanced imaging using cone-beam computed tomography, referral to experienced surgeons, modified surgical approaches, or consideration of coronectomy when appropriate. Careful surgical planning aimed at minimizing operative time and unnecessary bone removal may further reduce the incidence of postoperative neurosensory complications and improve patient outcomes.

Risk mitigation strategies

Based on the present findings, patients with multiple risk factors should undergo careful preoperative assessment and individualized surgical planning. When indicated, cone-beam computed tomography may aid in evaluating the relationship between the impacted tooth and the inferior alveolar canal. During surgery, meticulous technique, minimization of unnecessary bone removal and operative time, and gentle tissue handling are recommended to reduce the risk of IAN and LN injury. Appropriate patient counselling and close postoperative neurosensory monitoring are also essential to optimize outcomes.

Limitations

This study had certain limitations. Its retrospective design is susceptible to selection bias and depends on the completeness and accuracy of clinical documentation. IAN and LN injuries were analyzed as a composite postoperative neurosensory outcome because the relatively small number of nerve injury events precluded reliable nerve-specific analyses; consequently, differences in their anatomical characteristics and mechanisms of injury could not be evaluated separately. Furthermore, the relatively small number of postoperative nerve injury events may have limited the precision and stability of the multivariable regression estimates, and the observed associations should therefore be interpreted with appropriate caution. The study was conducted at a single institution, which may limit the generalizability of the findings. In addition, surgeon-related variables, including operator experience, flap design, instrumentation, and individual surgical technique, were not consistently available in the retrospective records and therefore could not be incorporated into the multivariable analysis, introducing the possibility of residual confounding.

Variations in surgeon experience, surgical techniques, and postoperative assessment protocols also could not be completely standardized. Moreover, postoperative neurosensory assessment was based on routine clinical documentation rather than a standardized objective sensory testing protocol, and therefore some degree of outcome misclassification cannot be excluded. Furthermore, detailed cone-beam computed tomography measurements were unavailable for all patients because advanced imaging was performed only when clinically indicated. Future prospective multicenter studies with standardized surgical protocols, objective neurosensory assessment methods, nerve-specific analyses, and larger sample sizes are warranted to validate these findings and develop robust risk prediction models for postoperative nerve injury following mandibular third molar surgery.

## Conclusions

Postoperative IAN and LN injuries after impacted mandibular third molar surgery are uncommon and predominantly transient. Advanced age, deep impactions, horizontal tooth angulation, and radiographic overlap with the inferior alveolar canal were identified as independent predictors of neurosensory deficits. Intraoperative factors reflecting greater surgical complexity, particularly prolonged operative time and extensive bone removal, further increase the risk. Comprehensive preoperative risk assessment and meticulous surgical planning are essential for minimizing nerve injury and optimizing patient outcomes.
